# Rare Association between Giant Right Ventricular Myxoma and Right Coronary Artery Tumour Blush with Complicating Pulmonary Tumour Embolism

**DOI:** 10.1155/2019/5873606

**Published:** 2019-04-15

**Authors:** Robin Yeong Hong Goh, Shreeja Mehrotra, Stefan Buchholz, Deepak Mehrotra, Allison Morton

**Affiliations:** ^1^Heart Care, Western Australia, Suite 21, St. John of God Bunbury Hospital, 700 Robertson Dr, College Grove, Western Australia, Australia; ^2^Department of Cardiothoracic Surgery, Heart and Lung Centre, Mount Hospital, 150 Mounts Bay Rd, Perth, Western Australia, Australia

## Abstract

Cardiac myxoma is a benign primary cardiac tumour which can present with nonspecific symptoms of right heart failure, syncope, exertional dyspnea, and pulmonary embolism. We describe a case of a right ventricular myxoma complicated with bilateral pulmonary embolism, with an incidental right coronary artery fistula but otherwise normal coronary anatomy on coronary angiogram. This case report emphasizes the importance of performing a transesophageal echo in the setting of pulmonary embolism to search for the origin of thrombus/tumour, and performing a comprehensive assessment is also necessary to rule out coronary artery disease, coronary artery fistula that may also represent a tumour blush.

## 1. Background

Cardiac myxoma is the most prevalent (50%) benign primary cardiac tumour [1], with 85% arising from the left atrium, 10% from the right atrium, and 5% from the ventricles [2]. Patients can present with constitutional symptoms (fever, lethargy, and weight loss), infection (endocarditis), or nonspecific symptoms of right heart failure, syncope, exertional dyspnea, and pulmonary embolism [3, 4].

## 2. Case Summary

**Figure 1:**
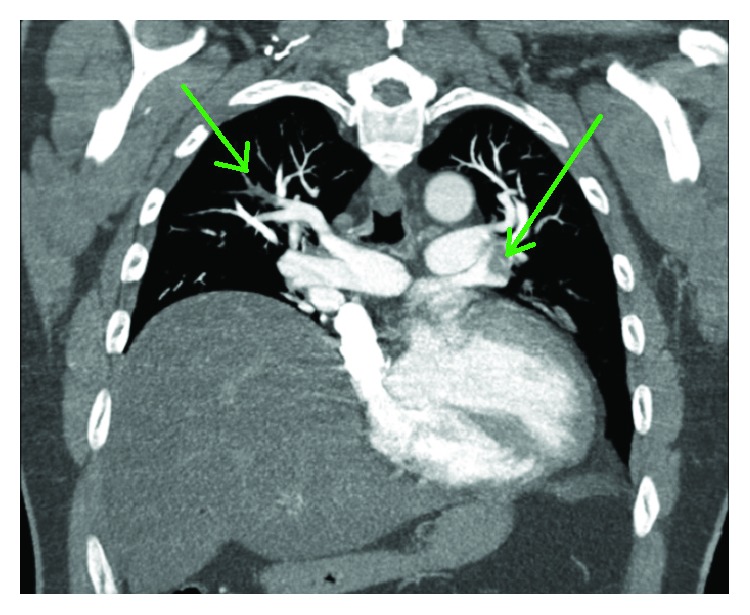
Computed tomography pulmonary angiogram revealed bilateral pulmonary emboli (green arrows).

**Figure 2:**
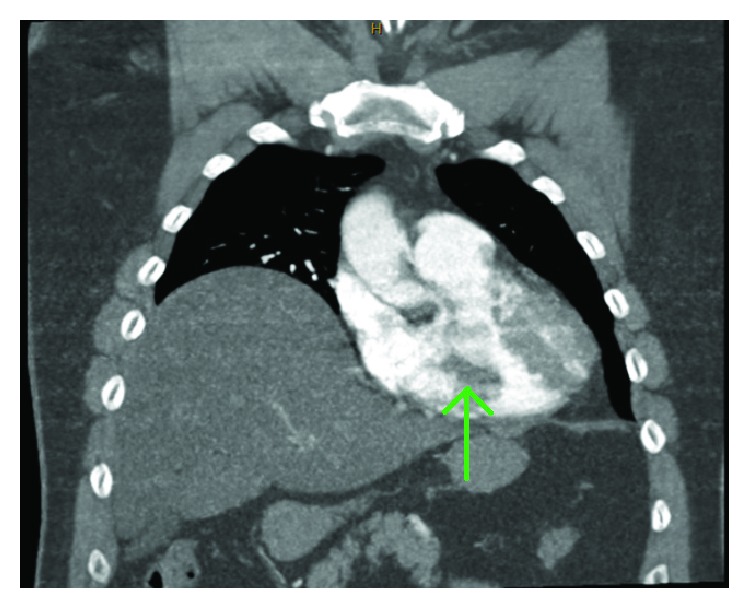
Computed tomography pulmonary angiogram revealed a broad-based filling defect in the right ventricle (green arrow), with incidental finding of preoperative raised right hemidiaphragm.

**Figure 3:**
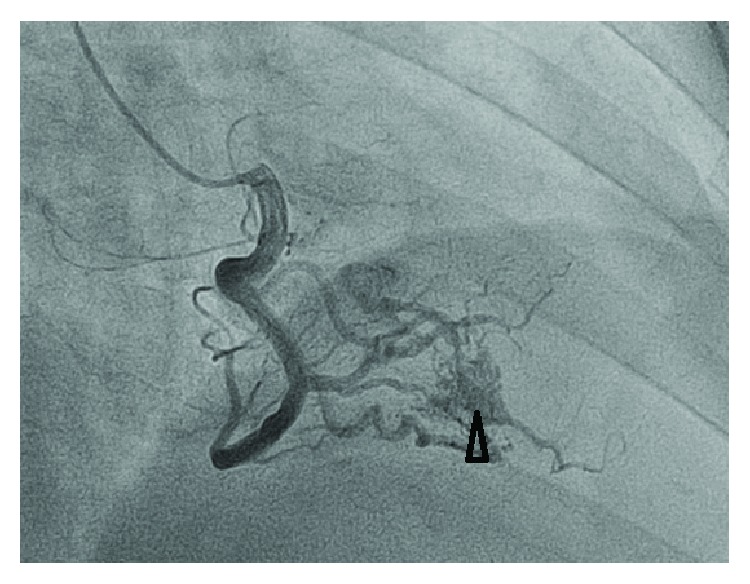
Coronary angiogram revealed no evidence of coronary artery disease, right coronary artery fistula, or tumour blush demonstrating blood supply to the base of tumour in the ventricular septum.

**Figure 4:**
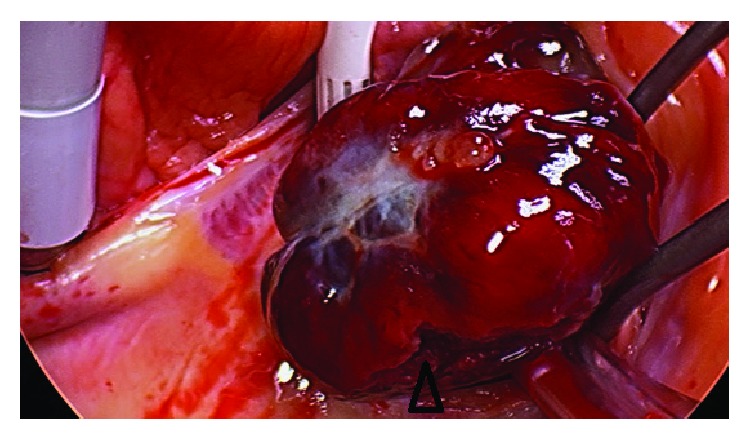
Intraoperative photograph of histologically proven large 5 cm × 4 cm right ventricular myxoma beneath the septal leaflet of the tricuspid valve. Area where part of myxoma have fragmented and embolized (arrowhead).

**Figure 5:**
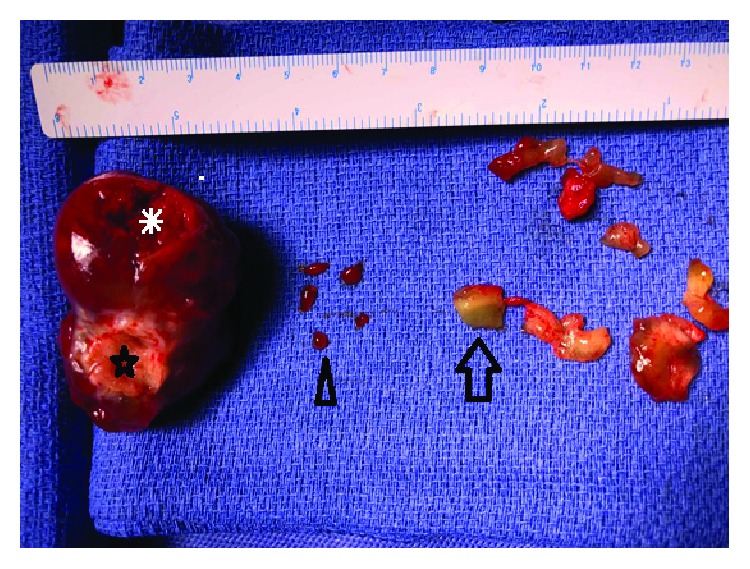
Pathology specimen of large 55 mm × 30 mm × 30 mm smooth right ventricular myxoma with attachment site to ventricular septum (star), part of myxoma that fragmented and embolized (asterisk), blood clot removed (arrowhead), and myxoma accessories (arrow).

**Figure 6:**
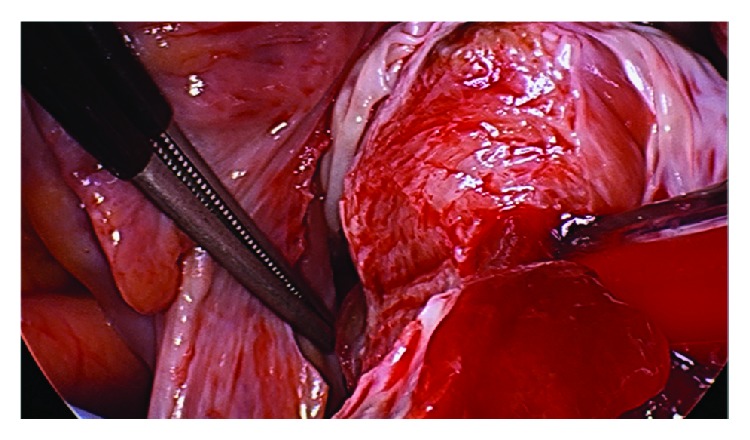
Right ventricular myxoma being excised and shaved off from ventricular septum. Note the origin of tumour beneath the septal and anterior leaflets of the tricuspid valve.

**Figure 7:**
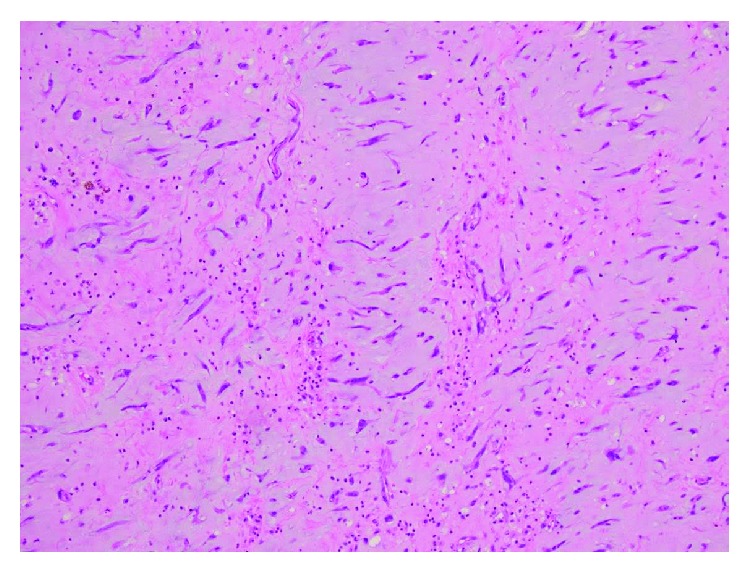
Histology of ventricular myxoma.

A 60-year-old man presented with pleuritic chest pain and shortness of breath on a background of exertional dyspnea associated with self-limiting central chest pain over the past eight years increasing over the past three weeks. Apart from having symptoms suggestive of obstructive sleep apnea, he is a nonsmoker and is in the low-risk group for developing thromboembolism with a Wells' score of zero on initial assessment. The patient also had no history of familial or recurrent myxoma or stigmata associated with Carney's complex [5]. In the work-up process, he was found to have no deep venous thrombosis (DVT), but computed tomography pulmonary angiogram (CTPA) showed large pulmonary embolism more on the left side (Figures 1 and 2). Transthoracic and transesophageal echo confirmed right ventricular filling defect (Figure 2), which initially was thought to be a right ventricular thrombus and was placed on intravenous heparin therapy. An angiogram revealed incidental distal right coronary artery fistula (Figure 3), without haemodynamic compromise, likely also represents “tumour blush.” During surgery on cardiopulmonary bypass (CPB), a myxoma arising from the right ventricular septum beneath the septal leaflet of the tricuspid valve, approximately 5 cm × 4 cm in size was identified (Figure 4) and removed without spilling and further fragmentation (Figure 5). The tumour was shaved from the septum without creating any ventricular septal defect (VSD) and protecting the tricuspid valve leaflets (Figure 6). A patent foramen ovale (PFO) was enlarged to create an atrial septal defect (ASD) to inspect the left-sided chambers of the heart, which was subsequently closed. The main pulmonary artery was opened to perform extensive tumour embolectomy, and tumour was retrieved in piecemeal. Histology of tumour and emboli both confirmed to be myxomatous in origin (Figure 7). The patient's postoperative recovery was uneventful. Postoperatively, he was anticoagulated with Clexane for 4 days and discharged with warfarin with a recommended therapeutic INR between 2 and 2.5 for his pulmonary embolism for a total of six months before reviewing in the follow-up consultation.

## 3. Discussion

Cardiac myxoma is associated with embolic phenomena in 30-40% of all cases [6]. Unlike in the left atrial myxoma where systemic arterial embolism is of concern, right-sided myxoma remains an important treatable cause to be ruled out in patients with idiopathic pulmonary embolism [6, 7]. Embolism is likely due to exposed nature and constant agitation of tumour which results in detachment of fragment either of the tumour or of the overlying thrombus.

High degree of suspicion of pulmonary embolism is necessary with consequent diagnosis. Missing the diagnosis can have fatal outcome. CTPA is gold standard for diagnosis and then search for thrombus/emboli origin. In current case, Doppler ultrasound of leg veins was negative for DVT, but echocardiogram confirmed the diagnosis of tumour in the right ventricle. These tumours have a tendency to prolapse into atrium and ventricle with each heartbeat. In this process, they can embolize or even obstruct the major atrioventricular valves with fatal outcome. Urgent surgery is mandatory for good outcome.

Embolization rates of patients with both the right and left cardiac myxomas vary between 25.3% [8] and 43% [9]. Studies have shown that morphology of cardiac myxoma is the most predictive factor of embolic events. Villous or papillary myxomas have multiple fine surface extensions that are more fragile and hence more easily embolized than round-shaped myxomas with a smooth surface [10, 11]. Other variables that were associated with embolic events include atrial fibrillation and small tumour size. Tumour size has been shown to be inversely related to the embolic event rate but related to the size of emboli. Friable myxomas tend to fragment and cannot grow large [6]. Conversely, even though it is rare for large cardiac myxomas to undergo total detachment, they still serve as a source for large embolic fragments [12]. Recurrence amongst nonfamilial cardiac myxomas is rare (Up to 3% after surgical resection), and time to recurrence for right-sided cardiac myxomas was reported between 36 and 72 months as compared to recurrence for left-sided cardiac myxomas which can recur earlier between 5 and 60 months [13].

In summary, this case report illustrates and supports early diagnosis to search for the cause of pulmonary embolism to reemphasize the importance of transesophageal echocardiogram in the diagnosis of origin of thrombus/tumour and early referral to surgery for good outcome as demonstrated in this case. Furthermore, long-term follow-up is essential to rule out the recurrence of such tumours. A high degree of suspicion is required to include tumour as an important entity in differential diagnosis of pulmonary embolism. Comprehensive assessment is necessary to rule out coronary artery disease, which is common in patients with cardiac myxomas [14], and other rare anomalies like coronary artery fistula [15]. In certain cases like this, it may represent tumour blush.
